# *Psoralea corylifolia* L. Attenuates Nonalcoholic Steatohepatitis in Juvenile Mouse

**DOI:** 10.3389/fphar.2017.00876

**Published:** 2017-11-30

**Authors:** Lishan Zhou, Jianqiao Tang, Xiaoli Xiong, Hui Dong, Juan Huang, Shunchang Zhou, Lingling Zhang, Huan Qin, Suqi Yan

**Affiliations:** ^1^Department of Integrated Traditional Chinese and Western Medicine, Wuhan Children's Hospital (Wuhan Maternal and Child Healthcare Hospital), Tongji Medical College, Huazhong University of Science and Technology, Wuhan, China; ^2^Institute of Integrated Traditional Chinese and Western Medicine, Tongji Hospital, Tongji Medical College, Huazhong University of Science and Technology, Wuhan, China; ^3^Department of Pathology, Wuhan Children's Hospital (Wuhan Maternal and Child Healthcare Hospital), Tongji Medical College, Huazhong University of Science and Technology, Wuhan, China; ^4^Center of Experimental Animals, Tongji Medical College, Huazhong University of Science and Technology, Wuhan, China; ^5^Department of Pediatrics, Integrated Traditional Chinese and Western Medicine Hospital of Wuhan, Wuhan, China; ^6^Laboratory, Wuhan Children's Hospital (Wuhan Maternal and Child Healthcare Hospital), Tongji Medical College, Huazhong University of Science and Technology, Wuhan, China

**Keywords:** *Psoralea corylifolia* L., nonalcoholic fatty liver disease, nonalcoholic steatohepatitis, PKC-α/NADPH oxidative signaling pathway, juvenile mouse

## Abstract

*Psoralea corylifolia* L. (PC) is a traditional Chinese herb used to treat yang deficiency of the spleen and kidney in pediatric disease. Recent studies have shown its liver protection and anti-oxidative effects. The aim of this study was to explore the effect and mechanism of PC on nonalcoholic steatohepatitis in juvenile mice. The juvenile mouse model of nonalcoholic fatty liver disease/nonalcoholic steatohepatitis (NAFLD/NASH) was established by being fed a high-fat diet in maternal-offspring manner. PC granules were prepared and the quality was assessed. The main components were identified by high performance liquid chromatography. Then, different dosages of PC were administered for 6 weeks. Homeostatic model assessment of insulin resistance, plasma liver enzymes, hepatic morphology, hepatic superoxide anion, and triglyceride/total cholesterol levels were examined. The changes of nuclear factor-κB (NF-κB) activity phosphatidylinositol 3 kinase (PI3K)/protein kinase B (Akt) and protein kinase C-α (PKC-α)/nicotinamide-adenine dinucleotide phosphate (NADPH) oxidase signaling pathways in hepatic tissues were also determined. Our data demonstrated that PC significantly improved liver dysfunction, liver triglyceride/total cholesterol accumulation and insulin resistance in juvenile NAFLD/NASH mice. PC also alleviated hepatic steatosis, inflammatory cell infiltration, and fibroplasia in the portal area. Additionally, PC inhibited the activation of NF-κB and the mRNA expression of inflammatory factors while enhancing PI3K/Akt signaling in hepatic tissues. PC could also reduce hepatic superoxide anion levels, and NADPH oxidase activity as well as p47^phox^ protein expression and PKCα activation in hepatic tissues. The results suggest that PC is effective in the treatment of NASH in juvenile mice. The mechanism may be related to the attenuation of hepatic oxidative stress through the PKC-α/NADPH oxidase signaling pathway.

## Introduction

Nonalcoholic fatty liver disease (NAFLD) is the most common chronic liver disease worldwide. NAFLD includes a broad spectrum of liver disease, ranging from simple steatosis to nonalcoholic steatohepatitis (NASH) and NASH can progress to liver cirrhosis and hepatocellular carcinoma. The epidemic of NAFLD is rapidly becoming a severe public health problem (Watanabe et al., 2015; Younossi et al., 2016). Recently, NAFLD has been recognized in children and is a major cause for referral to pediatric gastroenterologists and hepatologists (Loomba et al., 2009; Mencin and Lavine, 2011; Anderson et al., 2015). Because pediatric NAFLD is closely related to metabolic syndrome and adult cirrhosis its epidemic attracts much attention (Alkhouri and Feldstein, 2012). In China, the prevalence of childhood obesity is also a major public concern (Ji, 2008; Guo et al., 2013). This may be attributed to the rise of middle-class families and the comprehensive opening of the “two-child” policy with the process of urbanization in China. It is reported that 68.2% of children who are obese exhibit a fatty liver (Fan, 2013; Zhu and Fan, 2013). Pediatric NAFLD has taken the place of chronic viral hepatitis to be a major health problem in China.

Unfortunately, to date, there are no approved therapies for children and adolescents with NAFLD other than lifestyle advice on diet and exercise (Zelber-Sagi et al., 2016; Fleet et al., 2017). However, several promising targets have been identified with the extensive investigation of the pathophysiological mechanism of NAFLD. Among these, the importance of oxidative stress (OS) in the pathogenesis of NAFLD has been well-documented (Spahis et al., 2017). Nicotinamide-adenine dinucleotide phosphate (NADPH) oxidase is a key enzyme which mediated specific OS in the development of NAFLD. NADPH derived reactive oxygen species (ROS) that not only induces oxidative damage but also serves as a second messenger leading to inflammation and insulin resistance (IR) (Paik et al., 2014). Therefore, NADPH oxidase specific antioxidant therapies may be promising in the treatment of NAFLD.

*Psoralea corylifolia* L. (PC), a traditional Chinese herb, is widely used to treat yang deficiency of the spleen and kidney in pediatric disease in China. Modern pharmacological studies have proved that PC exerts beneficial effects on adult diseases such as cancer and osteoporosis (Chopra et al., 2013; Seo et al., 2014). In a recent study, its liver protection and anti-oxidative effects have also attracted attention (Seo et al., 2016). Our previous studies have found that PC can alleviate the kidney OS of rats with diabetic nephropathy, and its mechanism is related to the inhibition of NADPH oxidase (Zhou et al., 2013). However, the effect of PC on pediatric NASH is rarely reported. Therefore, the aim of this study was to explore the effect of PC on pediatric NASH in juvenile NAFLD mouse and investigate its possible mechanisms.

## Materials and methods

### Animals

Male (*n* = 5) and female (*n* = 15) C57BL6J mice, age 56 to 63 days, obtained from the Institute of Laboratory Animal Sciences, CAMS&PUMC, were used in this study (Certification: no.11401300045242). The mice were maintained at ambient temperature (22 ± 1°C) with a 12:12 h light-dark cycle and free access to water and food (Barrier system, Center of Experimental Animals, Tongji Medical College, Huazhong University of Science and Technology. Certification: no.00142666). All experiments were approved by the animal ethics committee of Wuhan Medical & Health Center for Women and Children, Huazhong University of Science and Technology (no. 2014010).

### Diets

The Laboratory Animal Center of Tongji Medical College of Huazhong University of Science and Technology provided a standard diet (35% flour, 20% soy meal, 20% corn meal, 15.5% bran, 0.5% bean oil, 5% fish meal, 2.5% bone meal, 1% dusty yeast, and 0.5% salt). High-fat diets D12451, 4.73 total kcal/g (45% kcal and 24% g fat, 20% kcal and 24% g protein, 35% kcal and 41% g carbohydrate), were purchased from Research Diets, Inc. (New Brunswick, NJ, USA).

### Herbal materials and assessment of the quality

*Psoralea corylifolia* L. concentrated granules were provided by Sanjiu Medical and Pharmaceutical Co. Ltd. (Shenzhen, China). One gram of PC granule equals to 20 g PC traditional Chinese herbals. High performance liquid chromatography (HPLC) was performed to identify the main chemical constituents in PC concentrated granules to assess the quality. *Psoralen* (P.N. 110739–201416) and *Isopsoralen* (P.N. 110738–201614) purchased from National Institutes for Food and Drug Control (Beijing, China), were taken as reference standards. HPLC was performed on Agilent 1260 system (USA) equipped with an Agilent ZORBAX Eclipse Plus C18 (250 × 4.6 mm, 5 μm). The mobile phase included methyl alcohol-waters (55:45, v/v) at a flow speed of 1.0 ml/min in condition of column temperature 30°C. The detection wavelength was 246 nm.

### Animal modeling and grouping

After adaptive feeding with a standard diet for 3 days, all female mice were randomized into the normal control group (*n* = 3) and model group (*n* = 12). All male mice were in the same randomization, with a ratio of 1:3 to female mice for mating. All male mice and female mice in the control group continued the standard diet. Female mice in four model groups were fed with high-fat diets 1 week before conception and during gestation and lactation. Litter size was standardized to four pups to guarantee that no litter was nutritionally biased. After weaning, the female offspring mice in the control group were selected and fed a standard diet: the normal control group (Control, *n* = 6). The female offspring mice in the model groups were selected and assigned to high-fat diets, generating four other groups: the juvenile NAFLD model group (Model, *n* = 6), low-dose PC treated group (PC-L, *n* = 6), high-dose PC treated group (PC-H, *n* = 6), vitamin E (Zhejiang Medicine Co., Ltd., Xinchang Pharmaceutical Factory, Shaoxing, China) treated group (VitE, *n* = 6). After 3 days, mice in the aforementioned treatment groups were administered corresponding therapy, PC-L (1.125 mg/g/d), PC-H (2.25 mg/g/d), or VitE (0.01 mg/g/d), intragastrically to 9 weeks of age. Oral gavage was performed once a day between 9:00 and 11:00 a.m. Mice in the normal control and juvenile NAFLD model group were administered the same volume of distilled water. Doses were adjusted to the body weight recorded once every 5 days.

### Sampling

All the offspring female mice were euthanized at the end of 9 weeks. Blood samples were obtained by orbital sinus puncture at the time of euthanization. The specimen serum was collected and stored at −20°C for analysis after centrifuging at 3,000 revolutions per minute for 30 min at 4°C. Livers were quickly removed by laparotomy and flushed with normal saline on ice. Samples from the left outside lobe of the liver were fixed in 4% paraformaldehyde solution for paraffin embedding. Samples from the left inside lobe of the liver were prepared for frozen sections. Samples from the right lobe and the caudate lobe of the liver were preserved at −80°C for the analysis.

### Calculation of insulin resistance index

Fasting blood glucose was measured by the hexokinase method using a GLU Kit (Shanghai Mind Bioengineering Co., Ltd., Shanghai, China). Fasting insulin was determined by enzyme-linked immunosorbent assay method using a Mouse Ins (Insulin) ELISA Kit (Elabscience Biotechnology Co. Ltd., Wuhan, China). Homeostatic model assessment of insulin resistance (HOMA-IR) was calculated according to the formula as follows: fasting blood glucose (mmol/L) × fasting insulin (mIU/L)/22.5.

### Biochemical analysis

Serum levels of alanine aminotransferase (ALT), aspartate aminotransferase (AST), and liver content of triglycerides (TG), and total cholesterol (TC) were measured using commercial reagents (Jiancheng Bioengineering Institute, Nanjing, China).

### Hepatic histology

The paraffin slides stained with hematoxylin and eosin (H&E) were observed under optical microscope to assess hepatocyte steatosis and cellular infiltrate. The paraffin slides stained with sirius red were observed under polarizing microscope (Axio Scope.1A, Carl Zeiss, Germany) to assess the collagen deposition of the liver. The following criteria were used for scoring hepatic histology. The Kleiner scoring system was used to evaluate the severity of NAFLD (Kleiner et al., 2005). An activity score was generated by adding the individual scores for three features: steatosis (<5% = 0, 5–33% = 1, 33–66% = 2, >66% = 3); ballooning (none = 0, few = 1, prominent = 2); and lobular inflammation (none = 0, <2 foci = 1, 2–4 foci = 2, >4 foci = 3). A score of <3 represents a mild nonalcoholic fatty liver; a score of 3 to 4 represents a moderate nonalcoholic fatty liver; and a score of 5 or more represents NASH.

### Quantitative real-time polymerase chain reaction analysis

Hepatic total RNA was extracted from the liver tissue with TRIzol reagent in accordance with the manufacturer's instructions. First, RNA purity and concentration were tested using a nucleic acid/protein analyzer (Thermo, USA). Second, the extracted total RNA (1 μg) was reverse transcribed using the PrimeScript™RRT Reagent Kit with gDNA Eraser (TaKaRa Company, Dalian, China) on a Mastercycler gradient PCR apparatus (Eppendorf Company, Germany). Next, the complementary DNA was kept at −20°C before PCR amplification. Next, real-time polymerase chain reaction (PCR) analyses were performed in 48-well optical PCR plates using SYBR® Premix Ex Taq™ (TaKaRa Company, Dalian, China) on an Applied Biosystems StepOne Real-Time PCR System (Thermo, USA). Finally, 2^−ΔΔCT^ was used for analyzing the data. Primer sequences are listed in Table 1.

**Table 1:** Real-time PCR primer sequences.

**Gene**	**Forward (5′ → 3′)**	**Reverse (5′ → 3′)**
**GAPDH**	5′-TGAAGGGTGGAGCCAAAAG-3′	5′-AGTCTTCTGGGTGGCAGTGAT-3′
**TNF-**α	5′-TCCCCAAAGGGATGAGAAGTT-3′	5′-GAGGAGGTTGACTTTCTCCTGG-3′
**IL-6**	5′-CTGGGAAATCGTGGAAATGAG-3′	5′-AAGGACTCTGGCTTTGTCTTTCT-3′
**IL-8**	5′-GGCCCAATTACTAACAGGTTCC-3′	5′-TGACTTCACTGGAGTCCCGTAG-3′
**MCP-1**	5′-CCCTACTATTCCTGATGGCACT-3′	5′-CTATGAGAAACCCACCACATCTG-3′

*Purchased from Wuhan Genecreate Bioengineering Co. Ltd., Wuhan, China. GAPDH, glyceraldehyde-3-phosphate dehydrogenase; IL-6, interleukin 6; IL-8, interleukin 8; MCP-1, monocyte chemotactic protein 1; TNF-α, tumor necrosis factor α*.

### Measurement of hepatic superoxide anion levels

Serving as an oxidant-sensitive probe, dihydroethidium (DHE) is widely used for the measurement of ROS. Ethidium and 2-hydroxyethidium, two products of DHE oxidation, bind to the nuclear DNA, thereby forming a strong red fluorescent complex (Zhou et al., 2013). Frozen sections of liver (6 μm) were incubated with DHE (5 mmol/L, Beyotime Institute of Biotechnology, Shanghai, China) in a dark container at 37°C for 30 min (Zhou et al., 2013) and then observed under an inverted microscope (IX51, Olympus, Japan).

### Measurement of liver NADPH activity

Liver homogenate was lyzed in mammal tissue protein extraction reagent. The extracted protein was then supplemented with a protease inhibitor cocktail and phenylmethylsulfonylfluoride (PMSF) (Sinopharm Chemical Reagent Co. Ltd., Shanghai, China). Next, the samples were centrifuged at 12,000 revolutions per minute for 15 min at 4°C. The supernatant was collected to quantify the protein concentration using a bicinchoninic acid protein assay kit (ASPEN Biotechnology Co. Ltd., Wuhan, China). Liver NADPH activity was measured using an NADPH Activity Quantification Kit (Genmed Scientifics Inc., Shanghai, China).

### Western blot analysis

Liver extracted proteins (40 μg) were mixed with sample buffer, boiled for 5 min, and subjected to 8 and 10% sodium dodecyl sulfate polyacrylamide gel electrophoresis gel graded from maximum to minimum of protein molecular weight (120 volts, 60–90 min). Separated proteins on the gel were transferred to 0.45 μm polyvinylidene fluoride membranes. The membranes were then blocked with 5% fat-free dry milk in Tris buffered saline with Tween (TBST) or 0.5% bovine serum albumin at room temperature for 1 h, followed by incubation with antibodies (Table 2) at 4°C overnight. The next day, after washing with TBST three times, the membranes were incubated with HRP-goat anti-rabbit/mouse antibody (ASPEN Biotechnology Co. Ltd., Wuhan, China) diluted 1:10,000 at room temperature for 30 min. After washing with TBST four times, immunoreactive proteins were detected using the chemoluminescence method (LiDE110, Canon, Japan). Finally, band densities were determined by AlphaEaseFC software and quantified as the ratio between OD-value of the target band to that of GAPDH.

**Table 2:** First antibody details.

**Antibody**	**Species**	**Manufacturer**	**Dilution ratio**
GAPDH	Rabbit	Abcam	1:10,000
Phosphorylated NF-κB p65 (S276)	Rabbit	Abcam	1:500
NF-κB p65	Rabbit	Abcam	1:1,500
PI3K p85	Rabbit	CST	1:3,000
Phosphorylated Akt (S473)	Rabbit	CST	1:1,000
Akt	Rabbit	CST	1:2,000
p47^phox^	Mouse	Santa	1:500
Phosphorylated PKC-α (S657 + Y658)	Rabbit	Abcam	1:500
PKC-α	Rabbit	Abcam	1:500

*Abcam (Hong Kong, China); CST (Danvers, MA, USA); Santa (California, USA). Akt, protein kinase B; GAPDH, glyceraldehyde-3-phosphate dehydrogenase; NF-κB, nuclear factor-κB; PI3K, phosphatidylinositol 3 kinase; PKC-α, protein kinase C -α*.

### Statistical analysis

All data were analyzed using SPSS19.0 statistical software. Statistical significance was assessed by one-way analysis of variance following Kolmogorov–Smirnov normality. With homogeneity of variances, significance between different groups was determined by least standard deviation (LSD) test; alternatively, the Games-Howell test was used. A probability of <0.05 was considered to be statistically significant.

## Results

### HPLC profile of PC concentrated granules

**Figure 1:**
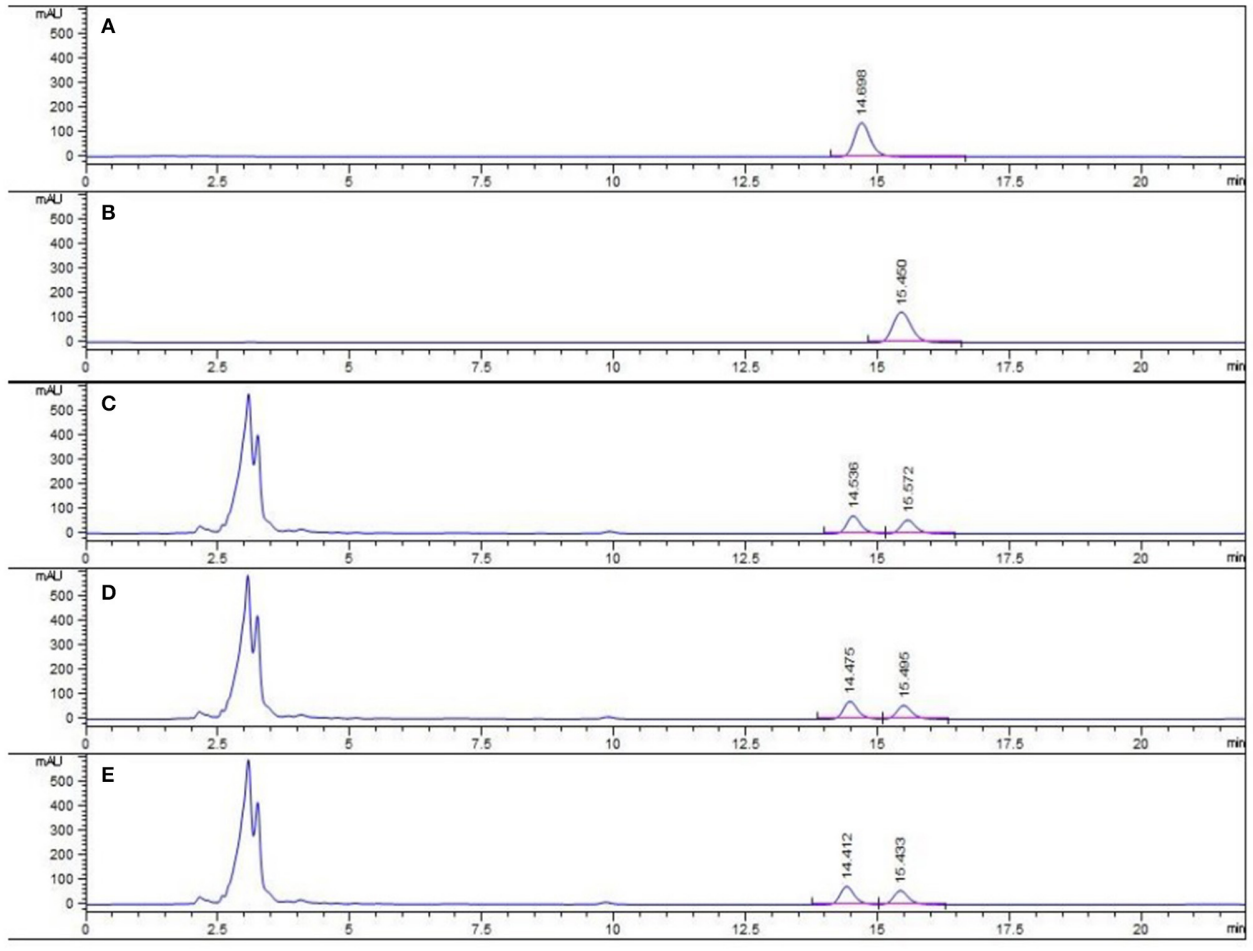
HPLC chromatogram of PC granule from three batches and reference standards. There were three batches of PC granule supplied by Sanjiu Medical and Pharmaceutical Co. Ltd. (lot: 1407001W, 1601002S, and 1601003S). **(A)** Standard with *Psoralea*; **(B)** Standard with *Isopsoralen*; **(C–E)** Three batches of PC granules samples.

The major active chemical constituents of PC are coumarins, flavonoids, and meroterpenes (Chopra et al., 2013; Zhang et al., 2016). According to Chinese Pharmacopoeia for the criterion of PC, coumarins: *psoralea* and *isopsoralen* were sleceted as markers for assessing the quality of the herb and some apropos preparations. HPLC of PC granules from three batches were shown in Figure 1. The result showed that the two main peaks were detected and identified by comparing the retention time with reference standards. *Psoralea* assay in three batches of PC granules were 7.035, 6.998, 6.730 mg/g, respectively. *Isopsoralen* assay in three batches of PC granules were 4.801, 4.858, 5.040 mg/g, respectively. The result verified that PC concentrated granules prepared from Chinese traditional medicinal PC and demonstrated that the three batch granules of testing data adhere to the Chinese Pharmacopoeia standard, as qualified samples can be used in the experiment.

### PC attenuated the progression of NASH in juvenile mice

#### Plasma liver enzymes levels

As shown in Table 3, untreated juvenile mice models presented with severe liver dysfunction. Both serum ALT and AST levels increased significantly in comparison with those of control mice (*p* < 0.01). However, treatment with different dose of PC and VitE reversed the increase in liver enzymes levels (*p* < 0.05, *p* < 0.01). Furthermore, a significant difference in serum ALT and AST levels was identified between high and low doses of PC, indicating a better improvement in liver dysfunction (*p* < 0.01).

**Table 3:** The effect of PC on the progression of NASH in juvenile mice.

**Group**	**Liver function**		**Liver TG and TC content**		**HOMA-IR**
	**ALT (U/L)**	**AST (U/L)**	**TG (mmol/gprot)**	**TC (mmol/gprot)**	
Control	43.92 ± 3.85	32.26 ± 3.02	2.02 ± 0.19	4.23 ± 0.28	3.69 ± 0.22
Model	91.59 ± 5.98^a^	58.33 ± 3.84^a^	5.98 ± 0.13^a^	7.43 ± 0.27^a^	8.73 ± 0.48^a^
PC-L	72.41 ± 3.35^c^	49.33 ± 2.75^b^	4.86 ± 0.27	6.45 ± 0.26^c^	7.08 ± 0.15^c^
PC-H	53.07 ± 3.52^c^^,^^d^	36.62 ± 2.61^c^^,^^d^	2.42 ± 0.26^c^	4.62 ± 0.17^c^^,^^d^	4.58 ± 0.56^c^^,^^d^
VitE	58.72 ± 2.55^c^	39.78 ± 1.58^c^	2.68 ± 0.48^b^	5.10 ± 0.36^c^	4.82 ± 0.48^c^

*Values are mean ± SD (n = 6)*.

a *p < 0.01 vs. the control group*,

b *p < 0.05*,

c *p < 0.01 vs. the model group*,

d *p < 0.01 vs. PC-L group*.

*HOMA-IR, Homeostatic model assessment of insulin resistance. ALT, alanine aminotransferase; AST, aspartate aminotransferase. TG, triglycerides, TC, total cholesterol*.

#### Liver TG and TC content

Compared with the juvenile mice in the control group, model mice presented with markedly higher liver TG and TC contents (*p* < 0.01). Mice in the PC-H and VitE groups exerted lower liver TG contents when compared with the model group (*p* < 0.01, *p* < 0.05). Mice in all treatment groups exerted lower liver TC contents when compared with the model group (*p* < 0.01). A significant difference in liver TC content was also identified between high and low dose of PC (*p* < 0.01). PC-H showed better effect on decreasing liver TG and TC contents than PC-L (Table 3).

#### Homeostatic model assessment of insulin resistance

Mice in the model group showed severe IR characterized by elevated HOMA-IR (*p* < 0.01). However, treatment with PC and VitE significantly decreased HOMA-IR compared with that of untreated model mice (*p* < 0.01). Furthermore, there is a significant difference in HOMA-IR between high and low doses of PC, indicating that PC-H exerted better effect on improving IR than PC-L (*p* < 0.01; Table 3).

#### Histological analysis

**Figure 2:**
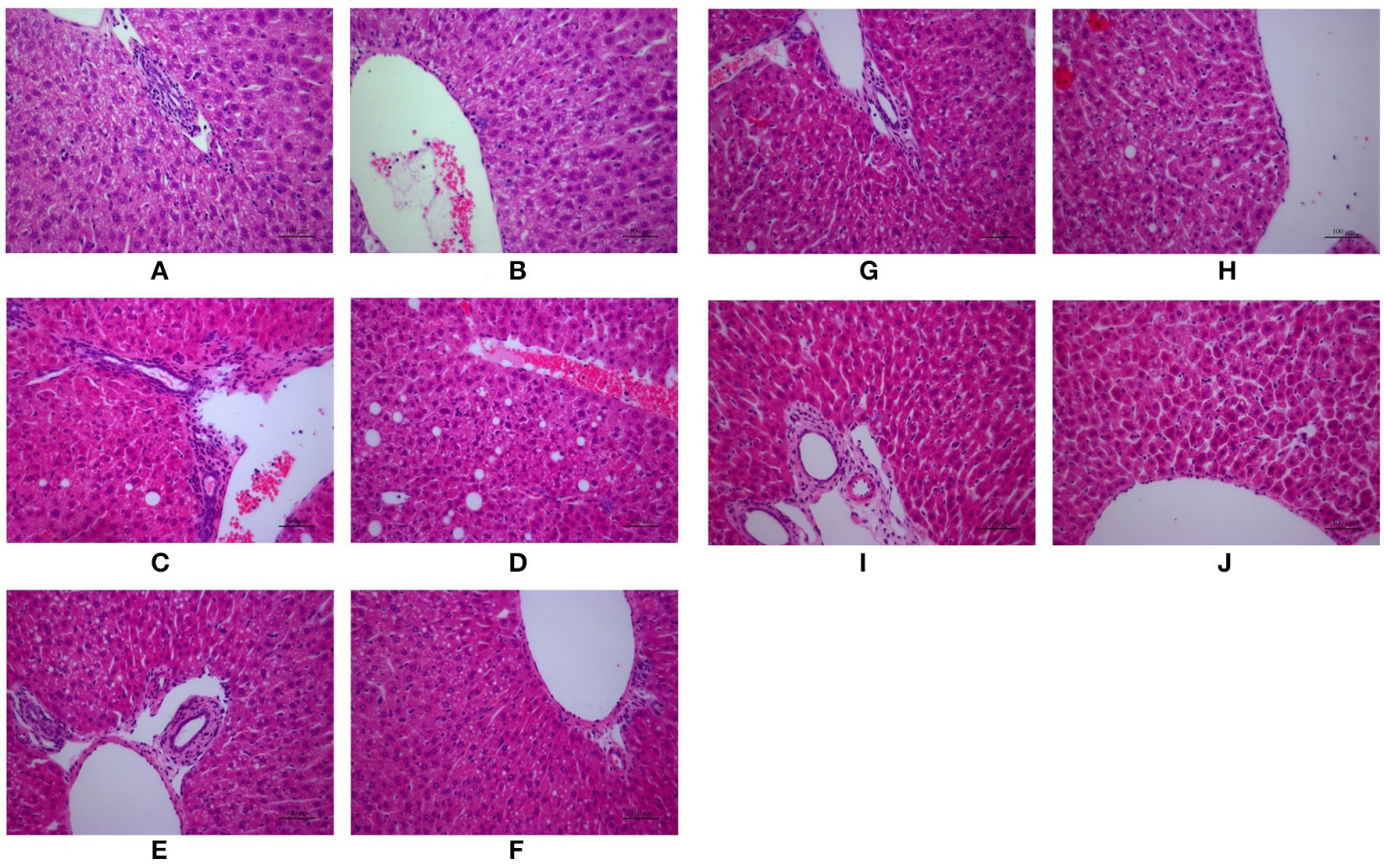
Morphologic photos of mice hepatic tissue. Hematoxylin and eosin (H&E) staining. Original magnification: ×200. **(A,B)** Control; **(C,D)** Model; **(E,F)** PC-L; **(G,H)** PC-H; **(I,J)** VitE.

As shown in Figure 2, H&E-stained liver tissues of mice in model groups showed hepatocyte steatosis, swelling, and degeneration; and fibroplasia and inflammation in the portal area with lymphocytic infiltrate. However, compared with the model group, the pathological changes of the liver in each treatment group were improved, and the high-dose PC group were the lightest. In addition, the severity of the NAFLD in the juvenile mice livers was assessed using the Kleiner scoring system (Kleiner et al., 2005). As shown in Table 4, the Kleiner score of mice in the control group was zero. Model mice achieved the highest score of 6.33, which indicates NASH with evidence of severe steatosis, ballooned hepatocytes, and inflammation. After the treatment, PC-H mice achieved the lowest score of 2, which indicates mild NAFLD. Both PC-L and VitE treatment offspring presented with moderate NAFLD, as indicated by scores of 4.33 and 3, respectively.

**Table 4:** Assessment of juvenile mice NAFLD/NASH severity in the liver tissues.

**Group**	**Steatosis**	**Ballooning**	**Inflammation**	**Activity score**	**Indication**
Control	0	0	0	0	Normal
Model	2.33	2	2	6.33	NASH
PC-L	1.33	1.33	1.67	4.33	Moderate NAFLD
PC-H	0.33	0.67	1	2	Mild NAFLD
VitE	0.67	1	1.33	3	Moderate NAFLD

*Values are mean (n = 3). Using the Kleiner scoring system (Kleiner et al., 2005). The average score for each histological characteristic in each group was used*.

### PC improved liver fibrosis in juvenile mice

**Figure 3:**
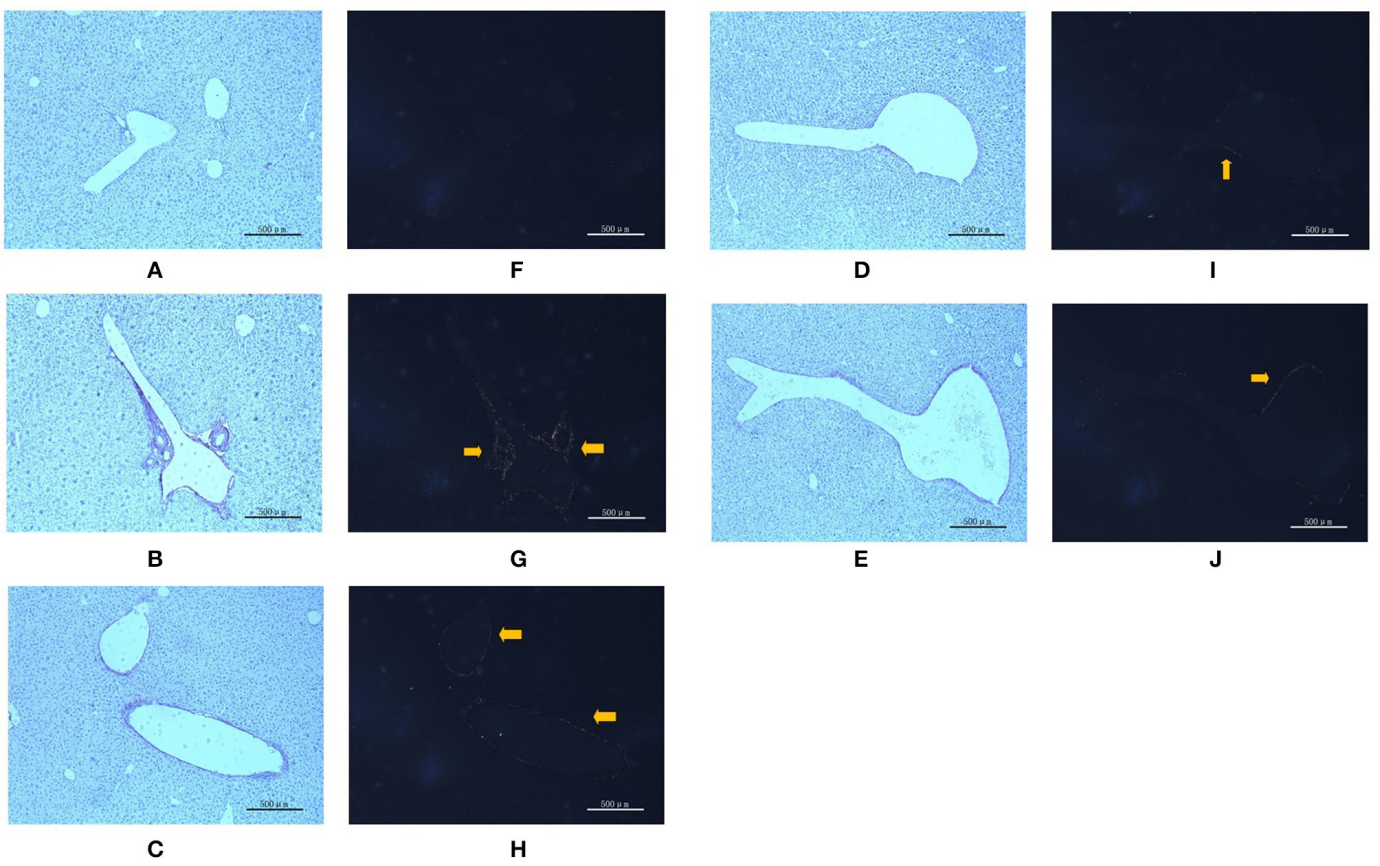
Sirius red staining in portal area from the mice in different groups. Original magnification: ×50. **(A,F)** Control; **(B,G)** Model; **(C,H)** PC-L; **(D,I)** PC-H; **(E,J)** VitE. **(A–E)** Using optical microscope. **(F–J)** Using polarizing microscopy (Arrows are pointing at the collagen deposition).

To verify liver fibrosis, we observed the collagen deposition in the portal and perisinusoidal areas by sirius red-stained liver sections (Figure 3). For the mice in the model groups, collagen deposition mainly occurred in the portal area of liver tissues. It manifested as a bright yellow light on polarizing microscopy, indicating portal fibrosis with type I collagen deposition. For the mice in the treatment groups, the type I collagen also deposited in the portal area of liver tissues. However, treatment with different dose of PC and VitE significantly weakened the level of yellow color in liver tissues of juvenile mice. In addition, the PC-H group presented weaker light than that of the PC-L groups.

### PC inhibited the activation of liver nuclear factor-κB in juvenile mice with NASH

#### Hepatic inflammatory factors expression

**Figure 4:**
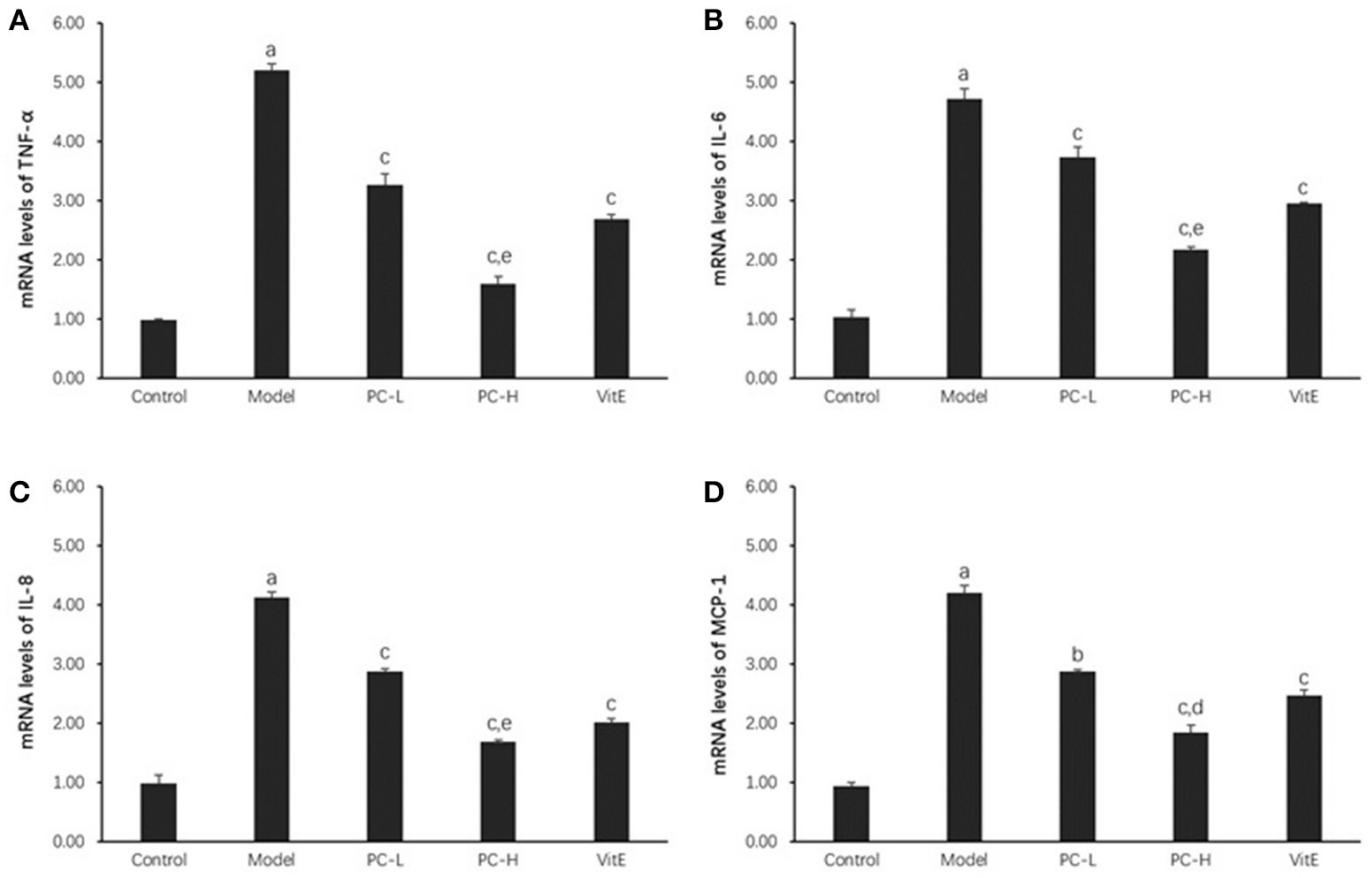
The effect of PC on the expression of liver inflammatory factors. Values are mean ± SD (*n* = 6). **(A)** TNF-α mRNA levels; **(B)** IL-6 mRNA levels; **(C)** IL-8 mRNA levels; **(D)** MCP-1 mRNA levels. The levels of mRNA were shown as the relative expression to the GAPDH. ^a^*p* < 0.01 vs. the control group, ^b^*p* < 0.05, ^c^*p* < 0.01 vs. the model group, ^d^*p* < 0.05, ^e^*p* < 0.01 vs. PC-L group. TNF-α, tumor necrosis factor α; IL-6, interleukin 6; IL-8, interleukin 8; MCP-1, monocyte chemotactic protein 1.

As shown in Figure 4, hepatic TNF-α, IL-6, IL-8, and monocyte chemotactic protein 1 (MCP-1) messenger RNA (mRNA) levels were markedly increased in the model group compared with those in the control group (*p* < 0.01). However, both PC and VitE treatment reduced the mRNA expressions of those liver inflammatory factors (*p* < 0.01, *p*<0.05). In addition, PC-H, better than PC-L, significantly decreased hepatic TNF-α, IL-6, and IL-8 mRNA expressions (*p* < 0.01) and hepatic MCP-1 mRNA expression (*p* < 0.05).

#### Hepatic NF-κB activity

**Figure 5:**
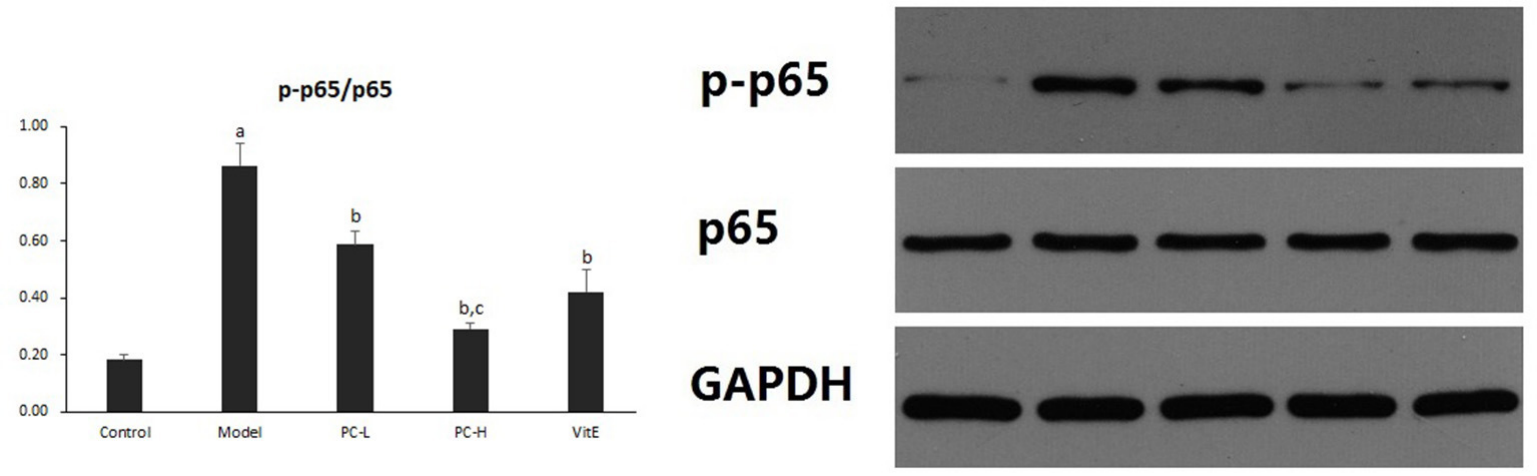
The effect of PC on the p-p65/p65 ratio in hepatic tissues. Values are mean ± SD (*n* = 6). ^a^*p* < 0.01 vs. the control group, ^b^*p* < 0.05 vs. the model group, ^c^*p* < 0.05 vs. PC-L group. p-p65: phosphorylated nuclear factor-κB p65; p65, nuclear factor-κB p65.

Hepatic NF-κB activity was shown as the ratio of phosphorylated nuclear factor-κB (NF-κB) p65 to NF-κB p65 (p-p65/p65) protein expression in liver tissues. As shown in Figure 5, it exhibited a marked elevation in p-p65/p65 ration in liver tissue of model mice (*p* < 0.01). The ratio was reduced after the treatment of PC or VitE (*p* < 0.05). Moreover, a reduction of p-p65/p65 was identified after high dose PC intervention when compared with low-dose PC (*p* < 0.05).

### PC downregulated liver PI3K-Akt signaling pathway in juvenile mice with NASH

#### Hepatic PI3K p85 expression

**Figure 6:**
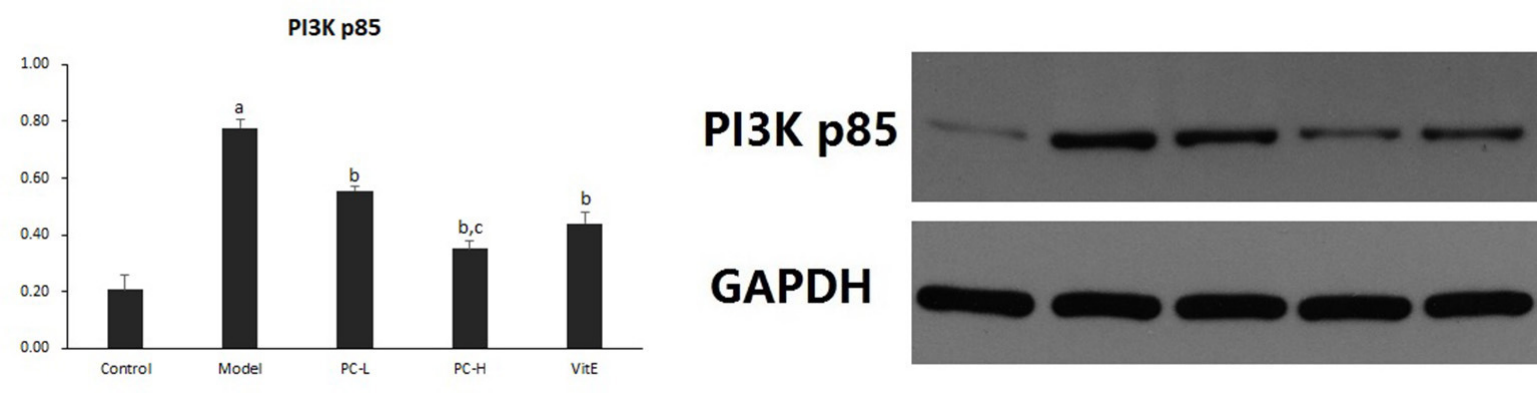
The effect of PC on the expression of PI3K p85 in hepatic tissues. Values are mean ± SD (*n* = 6). ^a^*p* < 0.01 vs. the control group, ^b^*p* < 0.01 vs. the model group, ^c^*p* < 0.01 vs. PC-L group. PI3K p85: phosphatidylinositol 3 kinase p85.

As shown in Figure 6, the expression of hepatic PI3K p85 protein level significantly was increased in model mice compared with that in control mice (*p* < 0.01). However, there was a significant reduction in the expression of PI3K p85 protein in all the treatment groups (*p* < 0.01). Additionally, PC-H treatment, better than PC-L treatment, remarkably decreased PI3K p85 protein expression (*p* < 0.01).

#### Hepatic Akt activity

**Figure 7:**
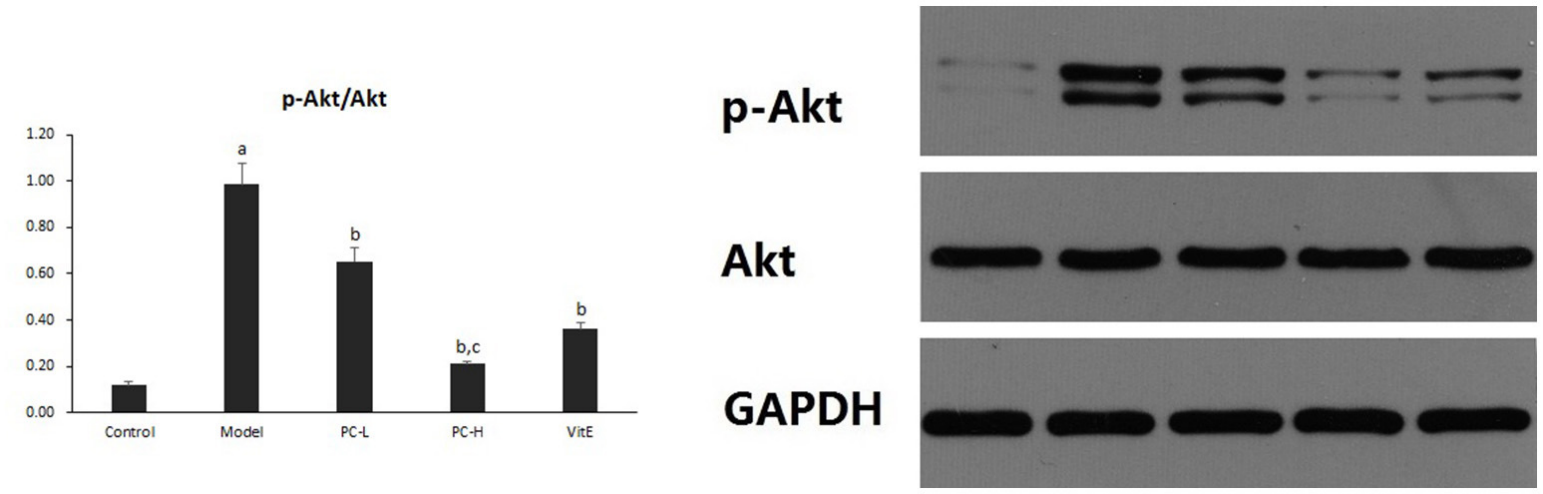
The effect of PC on the p-Akt/Akt ratio in hepatic tissues. Values are mean ± SD (*n* = 6). ^a^*p* < 0.01 vs. the control group, ^b^*p* < 0.01 vs. the model group, ^c^*p* < 0.01 vs. PC-L group. p-Akt, phosphorylated protein kinase B; Akt, protein kinase B.

Hepatic Akt activity was shown as the ratio of phosphorylated Akt to Akt (p-Akt/Akt) protein expression in liver tissues. As shown in Figure 7, it exhibited a marked elevation in p-Akt/Akt (*p* < 0.01) in liver tissues of model mice. The ratio was significantly reduced after the treatment of PC or VitE (*p* < 0.01). Moreover, a significant reduction of p-Akt/Akt was identified after high-dose PC intervention when compared with low-dose PC (*p* < 0.01).

### PC inhibited liver PKC-α/NADPH oxidase signaling pathway in juvenile mice with NASH

#### Hepatic superoxide anion production

**Figure 8:**
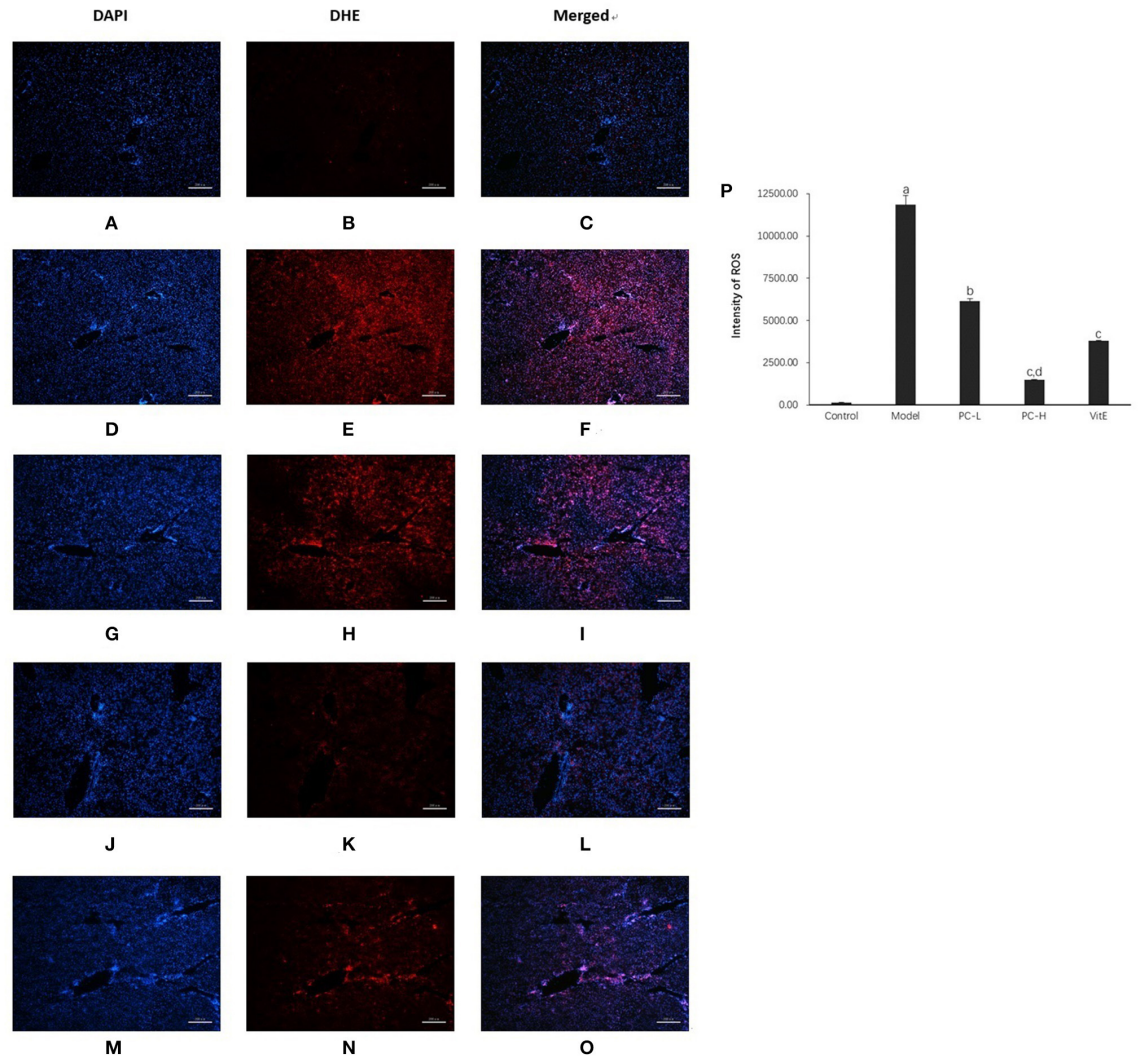
Dihydroethidium staining in the liver from the mice in different groups. Original magnification: ×40. **(A–C)** Control; **(D–F)** Model; **(G–I)** PC-L; **(J–L)** PC-H; **(M–O)** VitE. **(A,D,G,J,M)** Visualization of the nucleus in the liver using DAPI stains. **(B,E,H,K,N)** Visualization of ROS in the liver using DHE stains. **(C,F,I,L,O)** The superimposed photos of different groups. **(P)** Intensities of DHE staining levels were compared. The fluorescence intensity was obtained by Image-Pro Plus (IPP 6.0) and quantified as integrated option density (IOD) value. Values are mean ± SD (*n* = 3, 3 fields per animal). ^a^*p* < 0.01 vs. the control group, ^b^*p* < 0.05, ^c^*p* < 0.01 vs. the model group, ^d^*p* < 0.01 vs. PC-L group.

As shown in Figure 8, compared with the control mice, a significant high level of DHE fluorescence was observed in hepatic tissues of model juvenile mice (*p* < 0.01), indicating increased superoxide anion production. However, treatment with PC and VitE reduced the level of DHE fluorescence in liver tissues of juvenile mice (*p* < 0.01, *p* < 0.05). A high dose of PC treatment, compared with a low dose of PC, presented a greater decrease in peroxide anion production in liver tissues of juvenile mice (*p* < 0.01).

#### Hepatic NADPH activity

**Figure 9:**
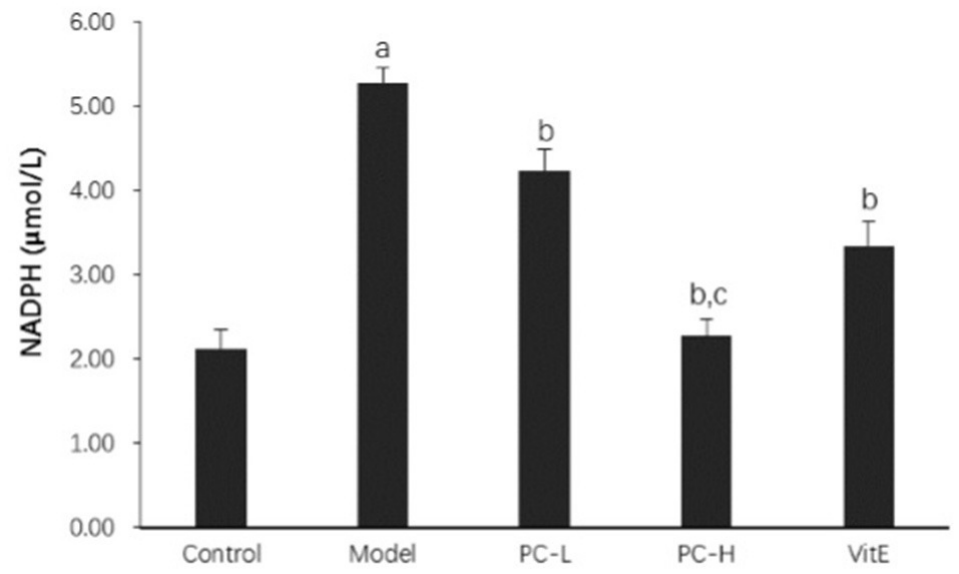
The effect of PC on the hepatic NADPH activity. Values are mean ± SD (*n* = 6). ^a^*p* < 0.01 vs. the control group, ^b^*p* < 0.01 vs. the model group, ^c^*p* < 0.01 vs. PC-L group. NADPH, nicotinamide-adenine dinucleotide phosphate.

As shown in Figure 9, the activity of hepatic NADPH was much higher in juvenile model mice than that in control mice (*p* < 0.01). After the treatment with PC and VitE, hepatic NADPH activity was significantly decreased (*p* < 0.01). Furthermore, a significant difference in hepatic NADPH activity was identified between different doses of PC, indicating a better hepatic NADPH decreasing activity of high doses of PC than that of low doses of PC (*p* < 0.01).

#### Hepatic p47^phox^ protein expression

**Figure 10:**
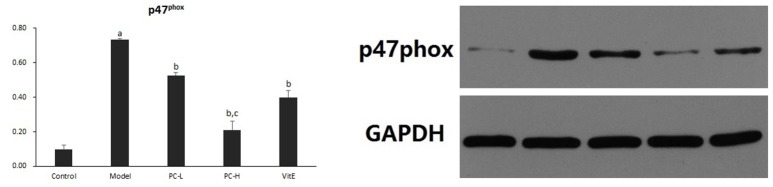
The effect of PC on the expression of p47^phox^ in hepatic tissues. Values are mean ± SD (*n* = 6). ^a^*p* < 0.01 vs. the control group, ^b^*p* < 0.01 vs. the model group, ^c^*p* < 0.01 vs. PC-L group.

As shown in Figure 10, hepatic p47^phox^ protein level was significantly increased in model mice compared with that in control mice (*p* < 0.01). However, there was a significant reduction in the expression of p47^phox^ protein in all the treatment groups (*p* < 0.01). Additionally, PC-H showed a better effect in decreasing p47^phox^ protein expression than PC-L treatment (*p* < 0.01).

#### Hepatic PKC-α activity

**Figure 11:**
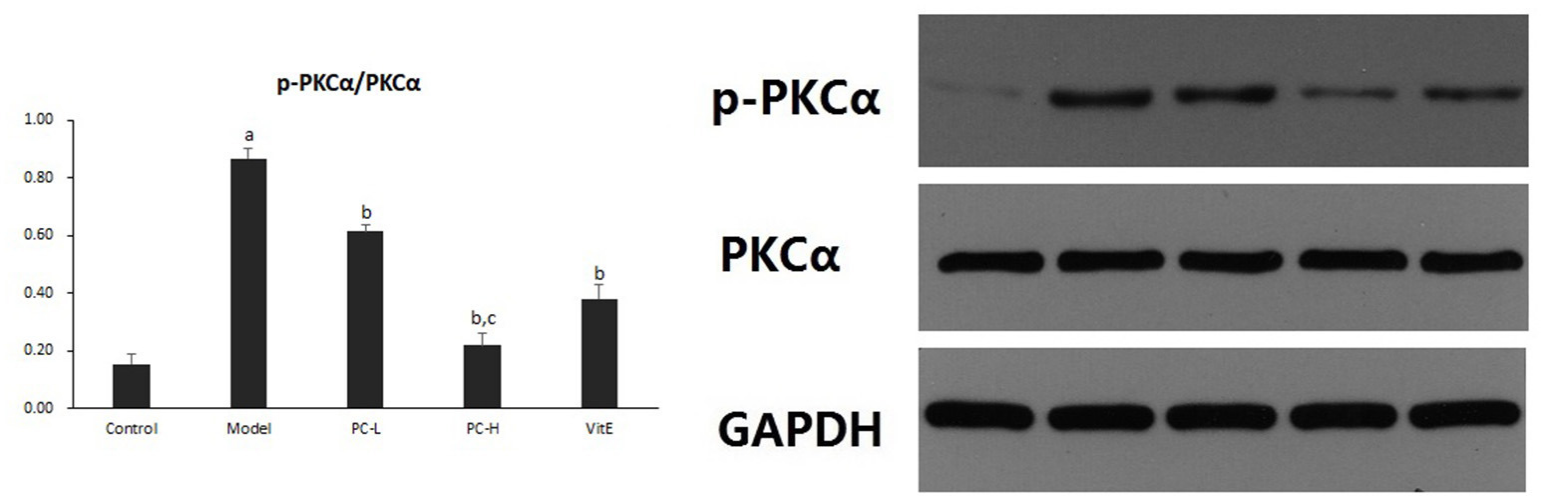
The effect of PC on the p-PKCα/PKCα ratio in hepatic tissues. Values are mean ± SD (*n* = 6). ^a^*p* < 0.01 vs. the control group, ^b^*p* < 0.01 vs. the model group, ^c^*p* < 0.01 vs. PC-L group. p-PKCα, phosphorylated protein kinase C-α; PKCα, protein kinase C-α.

Hepatic PKC-α activity was shown as the ratio of phosphorylated PKC-α to PKC-α (p-PKCα/PKCα) protein expression in liver tissues. As shown in Figure 11, it exhibited a marked elevation in p-PKCα/PKCα in liver tissue of model mice (*p* < 0.01). The ratio of p-PKCα/PKCα was significantly reduced after the treatment of PC or VitE (*p* < 0.01). Moreover, high-dose PC intervention showed a significant reduction of p-PKCα/PKCα when compared with low-dose PC (*p* < 0.01).

## Discussion

The traditional Chinese medicinal herb *P. corylifolia* L. (PC) is a hotspot in modern pharmacological research (Chopra et al., 2013). Thus far, the multiple pharmacological activities of PC have been discovered, especially its anti-osteoporosis and sex hormone-like effects (Li et al., 2014; Weng et al., 2015). However, the therapeutic effects of PC on metabolic diseases as fatty liver disease have rarely been reported and the mechanism is not clear.

The NASH model of juvenile mouse can be successfully established with a maternal-offspring high-fat diet feeding method (Bruce et al., 2009; Williams et al., 2014; Zhou and Pan, 2015). And a gender difference in the pathology progress was reported: only female presents hepatic oxidative stress (Marin et al., 2016). Hence, in our study, the female model mice were chosen and sampled before body maturation (9 weeks; Tang and Kong, 2006). Before adulthood, the juvenile mice manifested the characteristics of NAFLD, ranging from liver dysfunction (ALT > AST), elevated liver TG and TC contents, and IR to distinct liver histological pattern, with the predominant damage in the portal area: increased portal inflammation and fibrosis (Schwimmer et al., 2005; Carter-Kent et al., 2009; Fleet et al., 2017). Further Kleiner score analysis confirmed that the phenotype of fatty liver in juvenile offspring model mice was NASH. PC could relieve liver dysfunction, IR, and the accumulation of liver TG and TC. With regard to liver morphology changes, PC also attenuated the histopathological abnormalities in hepatic tissue in juvenile mice. Moreover, a high dose of PC was better in the aforementioned effects than a low dose of PC. These results suggest that PC can improve NASH progression in juvenile mice and the therapeutic effect is possibly dose dependent.

We also evaluated liver fibrosis by H&E staining and sirius red staining. We observed fibroplasia in the portal area of model juvenile mice. The coloration proved that the main type of collagen deposition might be positively related to type I collagen. However, the coloration level noticeably decreased after PC or VitE treatment. The high dose of PC showed the weakest light. These results indicate that PC may be effective on ameliorating liver fibrosis process in NASH.

The pathogenesis of NAFLD involves OS, IR, and inflammation. Each of these elements is fueled by the other and each promotes the other, thereby forming a vicious circle (Tilg and Moschen, 2010). Moreover, in the development and progression of NAFLD/NASH, OS, the real “first hit,” appears as the most crucial pathological event during NAFLD development and the hallmark between simple steatosis and NASH manifestation (Spahis et al., 2017), contributing to the aggravation of liver fibrosis process (Ratziu et al., 2010). Hence, this study further investigates the mechanisms of PC from the aforementioned three aspects and pays more attention to probes into the OS situation.

First, we evaluated the changes of the NF-κB signaling pathway to elucidate the anti-inflammation mechanisms of PC. We found that NF-κB activity was significantly increased in liver tissue of juvenile model mice accompanied by the increase in the expression of NF-κB downstream inflammatory factors in liver tissue. These inflammatory factors involved inflammatory cytokines (TNF-α and IL-6) and chemokines (IL-8 and MCP-1). Treatment with PC decreased NF-κB activity and TNF-α, IL-6, IL-8, MCP-1 mRNA expressions. The effects of high dose were also superior to that of low dose. These results suggest that the protection mechanisms of PC against juvenile NASH may be related to reducing inflammation by inhibiting hepatic NF-κB signaling pathway.

Second, to explicate the mechanism of PC at attenuating IR, we estimated the changes of the hepatic PI3K-Akt signaling pathway. It is well known that the upregulation of the PI3K-Akt pathway is of benefit to IR improvement. Nevertheless, our study validated that the protein expression of PI3K p85 was significantly increased in liver tissue of juvenile model mice, accompanied by the increase in Akt activity in liver tissue. Treatment with PC decreased hepatic PI3K p85 protein expression and p-Akt/Akt. Interestingly, this paradox has also been reported in some studies (McKee et al., 2015; Pisonero-Vaquero et al., 2015; Bankoglu et al., 2016). Actually, it is noteworthy that the AKT, a major downstream target of the PI3K, affects lots of downstream signaling pathways which involvement of metabolism, cell growth, and cell survival (Sheppard et al., 2012; Matsuda et al., 2013). The PI3K-Akt signaling pathway strongly influences hepatic stellate cells proliferation, the main collagen-producing cells in liver, leading to hepatic fibrosis (Heinrichs et al., 2011). These results suggest that the protection mechanisms of PC against fibrosis rather than IR may be related to downregulate the hepatic PI3K-Akt signaling pathway.

Third, we investigated the changes of OS situation. Excessive ROS generated by OS induces oxidative damage by peroxidation of the biomacromolecule and interferes with hepatic cell signal transduction by serving as a second messenger (Brandes and Kreuzer, 2005). To calculate superoxide anion production, liver DHE staining was performed. The appearance of superoxide can change DHE into ethidium bromide, which binds to DNA and exhibits red fluorescence in the nucleus (Gracia-Sancho et al., 2008; Vazquez-Chantada et al., 2013). In our study, a massive increase in superoxide anion production was identified in the young model offspring mice. However, a high dose of PC treatment, compared with a low dose of PC and VitE, showed the maximum decreasing of superoxide anion production. These findings further confirm the beneficial effect of PC on improving OS in juvenile NASH.

It is widely believed that the mechanism of anti-oxidant activity in PC involves scavenging ROS and inhibiting ROS generation (Chopra et al., 2013; Zhang et al., 2016). Finally, we assessed the changes of gene expression involved in the production of ROS. Among the multiple sources responding to chronic liver injury, ROS derived from NADPH oxidase is quite important to the development of NAFLD/NASH and hepatic fibrogenesis (Paik et al., 2014; Crosas-Molist and Fabregat, 2015). Our previous studies also confirmed that PC was effective in the treatment of diabetic nephropathy in rats and the underlying mechanism might be related to the attenuation of renal oxidative stress via PKC-α/NADPH oxidase signaling pathway (Zhou et al., 2013). Thus, the examination of the activity of NADPH oxidase and the expression of its upstream gene including PKC-α and p47^phox^ (Crosas-Molist and Fabregat, 2015) were carried out in the current study. We found that PC significantly inhibited NADPH oxidase activity and p47^phox^ protein expression in hepatic tissues of juvenile NASH mice. PKC-α activity was also decreased after PC treatment. PC at high dose was superior to PC at low dose in attaining the aforementioned outcomes. These results demonstrated that the inhibition of PC on ROS production might be related to the downregulation of PKC-α/NADPH oxidase signaling pathway in hepatic tissues.

In summary, we concluded that PC could improve the progression of NASH and ameliorate liver fibrosis in juvenile mice. The mechanism may be related to reduce inflammation by the NF-κB signaling pathway as well as inhibiting cells proliferation by the PI3K-Akt signaling pathway and decreasing OS through the PKC-α/NADPH oxidase signaling pathway. In addition, PC exhibits a dose dependent efficacy.

## Author contributions

LisZ and SY conceived and designed the study. LisZ, JT, XX, JH, SZ, LinZ, and HQ performed the experiments. LisZ and HQ wrote the paper. LisZ and HD reviewed and edited the manuscript. All authors read and approved the manuscript.

### Conflict of interest statement

The authors declare that the research was conducted in the absence of any commercial or financial relationships that could be construed as a potential conflict of interest. The reviewer DC and handling editor declared their shared affiliation.
